# Negative Impact of the COVID-19 Pandemic on Kidney Disease Management—A Single-Center Experience in Romania

**DOI:** 10.3390/jcm11092452

**Published:** 2022-04-27

**Authors:** Adrian Vasile Mureșan, Eliza Russu, Emil Marian Arbănași, Réka Kaller, Ioan Hosu, Eliza Mihaela Arbănași, Septimiu Toader Voidăzan

**Affiliations:** 1 Clinic of Vascular Surgery, Mures County Emergency Hospital, 540136 Targu Mures, Romania; muresanadi@yahoo.com (A.V.M.); eliza.russu@umfst.ro (E.R.); 2 Department of Surgery, George Emil Palade University of Medicine, Pharmacy, Science, and Technology of Targu Mures, 540136 Targu Mures, Romania; 3 Department of Nephrology, Mures County Emergency Hospital, 540136 Targu Mures, Romania; ioanhosu68@yahoo.com; 4 Faculty of Pharmacy, George Emil Palade University of Medicine, Pharmacy, Science, and Technology of Targu Mures, 540136 Targu Mures, Romania; arbanasi.eliza@gmail.com; 5 Department of Epidemiology, George Emil Palade University of Medicine, Pharmacy, Science, and Technology of Targu Mures, 540136 Targu Mures, Romania; septimiu.voidazan@umfst.ro

**Keywords:** nephrology, COVID-19, pandemic, chronic kidney disease, acute kidney insufficiency

## Abstract

Background: The evolution of the COVID-19 pandemic affected healthcare systems worldwide. The patients with chronic kidney disease (CKD), diabetes, and cardiovascular disease were most affected and had an unfavorable outcome. Methods: We examined the activity of the Nephrology Department from Târgu-Mureș County Emergency Hospital retrospectively, comparing two periods: June 2020–November 2021 (COVID-19 period) and June 2018–November 2019 (non-COVID-19 period). Results: In the COVID-19 period, there were fewer one-day hospitalizations registered, 77.27% more dialysis catheters were installed, and 43.75% more arteriovenous fistulas were performed. An overall increase in the number of patients requiring dialysis during the pandemic was recorded, as of the number of dialysis sessions performed. Moreover, we observed a statistically significant increase in the number of dialysis sessions per patient and a statistically significant increase in the number of hospitalization days in the pandemic interval. Acute kidney injury (AKI) and urosepsis were the diagnoses that increased the most among in-patients during the pandemic, while all other nephrology diagnoses decreased. Conclusions: The COVID-19 pandemic accelerated kidney pathology and worsened the outcomes of nephrology patients in our center. The number of chronic and patient’s access to one-day hospitalization decreased in order to minimalize the exposure and the risk of infection. In contrast, the need for emergency dialysis increased significantly.

## 1. Introduction

The severe acute respiratory syndrome coronavirus 2 (SARS-CoV-2), caused by the Coronavirus 19 Virus (COVID-19), was first diagnosed in December 2019 in Wuhan, China [1,2,3]. It spread globally in an alert rhythm, which led to the official declaration of the pandemic on 11th of March 2020 by the World Health Organization (WHO) [4]. Consequently, WHO recommended a set of restrictions to avoid spreading the virus, such as wearing masks, quarantining the people who tested positive with mild symptoms, and isolating the contacts.

The first confirmed case in Romania was declared on 26 February 2020 in Gorj county [5]. Until 14 March 2020, our country registered 100 confirmed cases, most of whom came from the countries severely afflicted by the pandemic [6].

**Figure 1:**
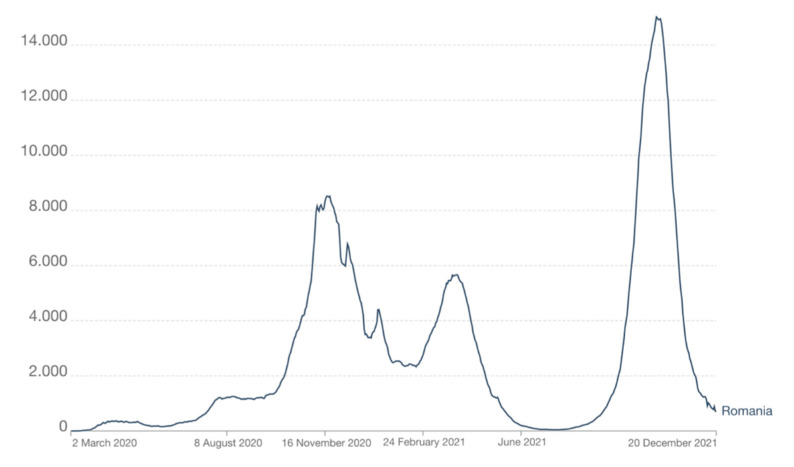
Evolution of the number of patients newly diagnosed with COVID-19 in Romania (Reprinted from Ref. [8]).

Until 24 December 2021, Romania registered 1801,081 confirmed cases and 58,463 fatalities [7]. The evolution of the pandemic has recorded three waves of maximum numbers of infected people, as follows: the first one from October 2020 until January 2021, where the first maximum number of cases in 24 h was on 18 November 2020 (10,269); the second wave took place in March–June 2021, with a maximum of infected patients recorded on 25 March 2021 (6651), and the third wave occurred in September–November 2021, with a maximum of 18,863 patients registered on 18 October 2021 (Figure 1) [8].

The evolution of the COVID-19 pandemic affected social life, daily activities, and healthcare systems. In the last one and a half years, numerous publications have dealt with the effect of the pandemic on medical activity, especially in fields such as oncology, cardiology, dermatology, plastic surgery, bariatric surgery, cardiac surgery, and transplant surgery [9,10,11,12,13,14,15,16,17,18,19,20,21]. Nationally, the medical system was subjected to specific measures to optimize the need to provide optimal and immediate care for the COVID-19 patients. Only emergencies were admitted during the lockdowns, chronic pathologies were rescheduled, and some hospitals were entirely transformed into COVID-19 facilities. The medical system was dichotomized to deal with emergencies and provide medical care for COVID-19 and non-COVID-19 patients.

In nephrology, the COVID-19 pandemic affected not only the patients already diagnosed with kidney impairment. For example, 30% of the patients with severe forms of COVID-19 developed acute kidney failure, and approximately 25–30% required hemodialysis [22,23,24,25,26]. The patients with chronic kidney disease (CKD), diabetes, and cardiovascular pathology were most affected and had an unfavorable outcome [27,28,29].

The Renal Association COVID-19 Database (ERACODA), founded in March 2020, deals with risk calculation and mortality rate assessment of the patients with CKD stage 5 [30] and kidney transplants. It reported a 28-day mortality of 21.3% for the COVID-19 confirmed patients among the kidney transplant recipients and 25% for the dialysis patients [31]. The overall activity of nephrology clinics and dialysis centers was affected by the lockdown measures [32,33].

The objective of this study was to analyze and compare the pandemic interval of June 2020–November 2021 with the pre-pandemic period June 2018–November 2019 and to draw conclusions regarding the effect of the pandemic on the overall activity of the Nephrology Department within the County Emergency Hospital Târgu-Mureș.

## 2. Materials and Methods

### 2.1. Study Design

We analyzed the activity of the Nephrology Department of Târgu-Mureș County Emergency Hospital retrospectively, comparing two periods of time: June 2020–November 2021 and June 2018–November 2019. The two analyzed periods were defined as the COVID-19 period (June 2020–November 2021) and the non-COVID-19 period (June 2018–November 2019). We have included in the study all the patients admitted to the Nephrology Department during the two specified periods, disregarding the type of admittance (chronic, emergency, one-day hospitalization). During the pandemic, the Nephrology Department of the County Emergency Hospital provided emergency care for COVID-19 and non-COVID-19 patients.

### 2.2. Data Collection

We recorded the number of one-day hospitalizations and the number of in-patients, and we collected demographic data, length of hospital stay, number of dialysis catheters used, number of arteriovenous fistulas (AVF) surgeries performed, number of dialysis sessions, and number of COVID-19 patients as independent data. Further, for the in-patients, we recorded the number of cases requiring intensive care and the number of deaths.

For the in-patients we stratified data according to the most encountered diagnoses, which were: Acute kidney insufficiency (AKI), CKD, urinary tract infection (UTI), acute interstitial nephritis (AIN), urosepsis, and other rare kidney diseases. We divided CKD into two categories: CKD stages 1–4, before dialysis, and end-stage kidney disease (ESKD).

### 2.3. Study Outcomes

The primary endpoints of the study was to compares the non-COVID-19 period and COVID-19 period regarding the numbers of one-day hospitalizations, dialysis catheter insertion, arteriovenous fistula surgeries, in-patients, intensive care unit (ICU) admittances, and deaths. As a secondary endpoint, we compared the number of in-patients admitted each month during both periods. We also compared the mean age, sex distribution, and hospital stay duration in a stratified manner, divided by in-patient main diagnosis. An additional endpoint is to present the outcomes of COVID-19 in-patients admitted to the Nephrology Department.

### 2.4. Ethical Approval

This study was conducted in accordance with the Declaration of Helsinki and approved by the Ethics Committee of Târgu-Mureș Emergency County Hospital, Romania (protocol code 31750, on 7 December 2021).

### 2.5. Statistical Analysis

Data are presented as the mean ± SD if normally distributed and median (Interquartile range) if non-parametrically distributed. Differences between groups were tested using two-tailed Student’s *t*-test or Mann–Whitney U test as appropriate for two-group comparisons. Categorical variables were compared with the χ2-test. All *p*-values are two-tailed, with *p* < 0.05 considered statistically significant. Statistical analysis was performed using SPSS for Windows version 22.0 (SPSS, Inc., Chicago, IL, USA).

## 3. Results

We included 1672 patients in the study. In the COVID-19 period, there were 77.27% more dialysis catheters installed, and 43.75% more AVF surgeries performed (Table 1). An overall increase by 14.05% (211 vs. 185) of the number of patients requiring dialysis during the pandemic was recorded, as well as a 32.44% (966 vs. 752) increase in the number of dialysis sessions performed. We also observed a statistically significant increase in the number of dialysis sessions per patient, counted in acute cases (mean value ± SD 4.72 ± 2.68 vs. 4.06 ± 3.08, *p* = 0.02). In contrast, there were only 289 one-day hospitalizations registered, 55.12% less than in the pre-pandemic period. We also registered a decrease in the number of in-patients during the pandemic, by 28.14% (309 vs. 430). We did not find statistically significant differences in sex distribution and mean patient age. However, a significant increase in the number of hospitalization days per patient in the pandemic interval was detected (*p* < 0.001). Moreover, an increase in the percentage of cases requiring intensive care (*p* < 0.001) and an increase in the rate of deaths (*p* < 0.001) were observed during the pandemic (Table 1).

During the pandemic period, 61 COVID-19 patients were admitted to the Nephrology Department. The mean age of these patients was 64.49 ± 12.46, ranging from 23 to 84 years, and the male to female ratio was 1.34. The average length of hospital stay was 13.16 days. Out of the 61 COVID-19 in-patients, 34 patients required intensive care and 29 deaths were registered (Table 2).

However, a significant increase in the number of hospitalization days per patient was detected in COVID-19 patients (*p* = 0.001). Further, an increase in the percentage of cases requiring intensive care (*p* < 0.001) and in the rate of deaths (*p* < 0.001) was observed in the COVID-19 patients (Table 2).

We analyzed the number of in-patients admitted each month. We registered a clear decrease of patient numbers during the pandemic, with one exception, May 2021 and 2019 (Table 3). The highest decreases in the number of patients were a 64.29% decrease in December 2020 compared to December 2018, followed by a 62.07% decrease in September 2021 compared to 2019, and a 60.87% drop in November 2021 compared to 2019. The first and third peaks led to the most significant decreases in the number of admitted patients (Table 3).

Next, we analyzed in-patient demographics divided by main diagnosis (Table 4). We registered a 52.77% drop in the numbers of UTI patients during the pandemic, without statistically significant differences regarding sex distribution or age, but with an increase of the average length of hospital stay (*p* = 0.01). A 52.03% decrease was observed in the number of patients with CKD stages 1–4, without any statistically significant differences regarding sex distribution, age, and number of hospitalization days. A significant drop by 36.8% could also be seen in the number of ESKD patients. These patients were significantly younger compared to the pre-pandemic interval (*p* = 0.03) and had longer hospitalization periods (*p* = 0.03).

The most significant decrease in the number of admitted patients was recorded for AIN patients, which decreased by 70.58% during the pandemic. The AIN in-patients were much older compared to the pre-pandemic period (average age 63.20 vs. 41.55 years, *p* = 0.005).

The number of patients admitted with urosepsis increased the most, by 76.92%, with no significant differences in age and length of hospitalization. Significantly more women were admitted with this diagnosis compared to men during the pandemic (*p* = 0.01). There was also a 41.7% increase in the number of AKI patients and the length of their hospitalization during the pandemic period (*p* = 0.03) (Table 4).

## 4. Discussion

The COVID-19 pandemic affected 279,330,865 people until 24 December 2021 and led to 5409,127 fatalities [7]. The possible complications are well known, i.e., severe cases developing multiple systems and organs failure (MSOF) [34,35]. The systematic review and meta-analysis published by Silver et al., including 54 studies and a total number of 30,639 patients, reported a 28% incidence of AKI among COVID-19 patients (95% CI, 22–34%). Of these patients, 9% required hemodialysis [36]. Gupta et al. found an even higher incidence (21%) of patients requiring hemodialysis for AKI related to COVID-19 infection [25].

The patients with chronic pre-pandemic kidney failure presented a high risk to develop AKI if infected. In the study of Cheng et al. on COVID-19 patients, a higher incidence of AKI was found among CKD patients compared with patients having normal kidney function pre-pandemic (11.9% vs. 4%) [37]. Similarly, in the meta-analysis on 1389 patients published by Henry et al., a significant positive association was found between pre-existing CKD and the severity of COVID-19 disease [OR 3.03 (95% CI 1.09–8.47)] [38].

In our study, we found that the number of patients requiring dialysis and the number of dialysis sessions were highly increased during the pandemic. The largest proportion of these cases was due to development of AKI, as the number of acute dialysis catheters inserted increased the most, by 77.27%. However, the number of AVF surgeries increased by 43.75%, suggesting that the COVID-19 pandemic also led to an accelerated progression of kidney failure towards terminal stages, requiring permanent AVFs.

In support of these data, there was a large increase in the number of in-patients with AKI and urosepsis during the pandemic, in stark contrast with the decrease of all other nephrological pathologies considered. These findings are primarily explained by the development of AKI and urosepsis in COVID-19 patients, some of which suffered MSOF. There was also a significant increase in the length of hospitalization of the ESKD and AKI patients, pathologies with the highest risk of acquiring the infection and having complicated evolutions. The mean age of ESKD patients was lower during the pandemic, suggesting that a younger patient population developed terminal kidney failure and required hospitalization.

We found that the drop in in-patient admissions was maintained throughout the pandemic, with the largest decreases clearly correlated with the three pandemic peaks. The decrease in the overall numbers of one-day hospitalizations and in-patients during the pandemic can be explained by two factors. On one hand, the fragile population of CKD patients were instructed to avoid hospital contact due to the risk of contracting COVID-19. On the other hand, as demonstrated by our data, the resources of the nephrology department have been redirected towards acute cases of COVID-19, AKI, and urosepsis, which required longer hospitalization periods. These patients were severely ill, as reflected by the substantial increase in the rates of ICU admissions and deaths during the pandemic among in-patients admitted to the department.

The strengths of this paper are the long period analyzed and the detailed analysis of interventions, dialysis sessions, diagnoses, length of stay, ICU admissions, and rates of death among patients admitted to the Nephrology Department. However, our study also has important limitations that have to be considered. Being a single center, we report a relatively small number of patients. Moreover, as patient management was influenced by the general regulations imposed by the Romanian Health Ministry related to the COVID-19 pandemic, the conclusions might not accurately reflect the experience of other nephrology departments worldwide and cannot be generalized.

## 5. Conclusions

The COVID-19 pandemic influenced renal disease management, affecting fragile patients and worsening their outcomes. The number of CKD patients admitted to the department decreased, to minimalize their exposure and the risk of infection. In contrast, the number of patients requiring acute hemodialysis increased substantially, mainly due to the COVID-19-related development of AKI. The need for AVFs also increased, suggesting a faster progression towards permanent hemodialysis. Our study brings an important piece of evidence concerning how the COVID-19 pandemic has affected disease progression and prognosis in nephrology patients, and how it has influenced the activity of the Nephrology Department of a large district hospital in Romania. Our findings can be used together with similar studies to gain a better understanding of the true impact of the pandemic on patient dynamics, prognosis, and healthcare systems worldwide.

## Figures and Tables

**Table 1:** Characteristics of the pre-pandemic and the pandemic periods.

	Non-COVID-19 Period 1 June 2018–30 November 2019	COVID-19 Period 1 June 2020–30 November 2021	Difference (%)	*p*-Value
Dialysis catheter insertion, no.	44	78	77.27%	-
AVF, no.	16	23	43.75%	-
Dialysis session, no.	752	996	32.44%	-
Dialysis patients, no.	185	211	14.05%	-
Dialysis session per patient, no. (mean ± SD)	4.06 ± 2.68	4.72 ± 3.08	-	0.02 ^b^
One day hospitalization patients	644	289	−55.12%	-
In-patients	430	309	−28.14%	-
Age mean ± SD (min-max)	65.92 ± 15.67 (20–96)	66.03 ± 15.04 (20–99)	-	0.92 ^a^
Male to female ratio	1.1	1.03	-	0.63 ^c^
Length of hospital stay (mean ± SD)	8.23 ± 4.63	9.74 ± 5.33	-	<0.001 ^b^
ICU admission, no. (%)	15 (3.48%)	49 (15.87%)	-	<0.001 ^c^
Deaths, no. (%)	79 (18.37%)	109 (35.27%)	-	<0.001 ^c^

AVF, arteriovenous fistulas; SD, standard deviation; ICU, intensive care unit; ^a^, student *t*-test; ^b^, Mann Whitney test; ^c^, chi square test.

**Table 2:** Characterics of COVID-19 period patients.

Variables	Non-COVID-19 Patients N = 248	COVID-19 Patients N = 61	*p*-Value
Age mean ± SD (min-max)	66.75 ± 14.37 (20–99)	64.49 ± 12.46 (23–84)	0.33 ^a^
Male to female ratio	0.96	1.34	0.24 ^c^
Length of hospital stay (mean ± SD)	8.85 ± 4.37	13.16 ± 9.91	0.001 ^b^
ICU admission, no. (%)	15 (6.04%)	34 (55.73%)	<0.001 ^c^
Deaths, no. (%)	80 (32.25%)	29 (47.54%)	0.02 ^c^

ICU, intensive care unit; SD, standard deviation; ^a^, student *t*-test; ^b^, Mann Whitney test; ^c^, chi square test.

**Table 3:** Numbers of in-patients admitted each month for both periods.

	Non-COVID-19 Period	COVID-19 Period	Difference (%)	Pandemic Peaks
	2018	2020		
June	27	22	−18.25%	
July	24	21	−12.5%	
August	30	25	−16.67%	
September	24	21	−12.5%	
October	27	26	−3.7%	
November	27	14	−48.15%	first peak
December	14	5	−64.29%	
	2019	2021		
January	25	18	−28%	
February	23	12	−47.83%	
March	26	21	−19.23%	second peak
April	18	13	−27.78%	
May	19	19	-	
June	25	20	−20%	
July	22	17	−22.73%	
August	24	19	−20.83%	
September	29	11	−62.07%	
October	23	16	−30.43%	third peak
November	23	9	−60.87%	

**Table 4:** In-patients diagnoses and characteristics for both periods.

	Non-COVID-19 Period 1 June 2018–30 November 2019	COVID-19 Period 1 June 2020–30 November 2021	Difference (%)	*p*-Value
UTI	36	17(2) *	−52.77%	-
Age mean ± SD (min-max)	65.11 ± 19.37 (22–89)	65 ± 19.12 (29–90)	-	0.98 ^a^
Male to female ratio	0.63	1.12	-	0.33 ^c^
Length of hospital stay (mean ± SD)	6.22 ± 2.15	9.29 ± 6.73	-	0.01 ^b^
CKD STG 1–4	123	59(6) *	−52.03%	-
Age mean ± SD (min-max)	71.67 ± 12.63 (32–96)	69.18 ± 13.58 (20–91)	-	0.23 ^a^
Male to female ratio	1.15	0.84	-	0.31 ^c^
Length of hospital stay (mean ± SD)	8.83 ± 4.19	9.37 ± 5.11	-	0.39 ^b^
ESKD	144	91(15) *	−36.8%	-
Age mean ± SD (min-max)	65.75 ± 10.85 (36–89)	62.57 ± 11.82 (25–89)	-	0.03 ^a^
Male to female ratio	1.44	1.11	-	0.34 ^c^
Length of hospital stay (mean ± SD)	8.15 ± 5.37	8.85 ± 4.68	-	0.03 ^b^
AIN	34	10(1) *	−70.58%	-
Age mean ± SD (min-max)	41.55 ± 16.05 (20–88)	63.2 ± 18.93 (25–85)	-	0.005 ^a^
Male to female ratio	0.17	0.66	-	0.08 ^c^
Length of hospital stay (mean ± SD)	6.26 ± 1.94	8.1 ± 3.84	-	0.16 ^b^
AKI	68	96(25) *	41.17%	-
Age mean ± SD (min-max)	69.85 ± 14.7 (21–95)	69.13 ± 15.23 (23–99)	-	0.76 ^a^
Male to female ratio	1.34	1.23	-	0.78 ^c^
Length of hospital stay (mean ± SD)	9.07 ± 4.79	10.65 ± 5.85	-	0.03 ^b^
UROSEPSIS	13	23(12) *	76.92%	-
Age mean ± SD (min-max)	59.23 ± 19.49 (23–83)	67.69 ± 15.94 (37–94)	-	0.19 ^a^
Male to female ratio	3.33	0.53	-	0.01 ^c^
Length of hospital stay (mean ± SD)	10.0 ± 5.08	11.39 ± 4.89	-	0.22 ^b^
OTHER	12	13(0) *	8.33%	-

UTI, urinary tract infection; CKD STG I-IV, chronic kidney disease stage 1 to 4; ESKD, end-stage kidney disease; AIN, acute interstitial nephritis; AKI, acute kidney insufficiency; SD, standard deviation; ^a^, student *t*-test; ^b^, Mann Whitney test; ^c^, chi square test; * The numbers in parentheses represent the number of COVID-19-positive in-patients for each diagnosis.
